# Long-lasting insecticidal nets no longer effectively kill the highly resistant *Anopheles funestus* of southern Mozambique

**DOI:** 10.1186/s12936-015-0807-z

**Published:** 2015-08-05

**Authors:** Katey D Glunt, Ana Paula Abílio, Quique Bassat, Helder Bulo, Allison E Gilbert, Silvie Huijben, Maria Nélia Manaca, Eusebio Macete, Pedro Alonso, Krijn P Paaijmans

**Affiliations:** ISGlobal, Barcelona Ctr. Int. Health Res. (CRESIB), Hospital Clínic, Universitat de Barcelona, Barcelona, Spain; National Institute of Health (INS), Ministry of Health, Maputo, Mozambique; Centro de Investigação em Saúde de Manhiça (CISM), Manhiça, Mozambique; Vector Control Reference Laboratory, National Institute for Communicable Diseases of the National Health Laboratory Service, Johannesburg, South Africa; National Directorate of Public Health, Ministry of Health, Maputo, Mozambique

**Keywords:** *Anopheles funestus*, *An. arabiensis*, Bioassays, Insecticide resistance, LLINs, Malaria vectors, Mosquito control, Pyrethroids

## Abstract

**Background:**

Chemical insecticides are crucial to malaria control and elimination programmes. The frontline vector control interventions depend mainly on pyrethroids; all long-lasting insecticidal nets (LLINs) and more than 80% of indoor residual spraying (IRS) campaigns use chemicals from this class. This extensive use of pyrethroids imposes a strong selection pressure for resistance in mosquito populations, and so continuous resistance monitoring and evaluation are important. As pyrethroids have also been used for many years in the Manhiça District, an area in southern Mozambique with perennial malaria transmission, an assessment of their efficacy against the local malaria vectors was conducted.

**Methods:**

Female offspring of wild-caught *Anopheles funestus s.s.* females were exposed to deltamethrin, lambda-cyhalothrin and permethrin using the World Health Organization (WHO) insecticide-resistance monitoring protocols. The 3-min WHO cone bioassay was used to evaluate the effectiveness of the bed nets distributed or available for purchase in the area (Olyset, permethrin LLIN; PermaNet 2.0, deltamethrin LLIN) against *An. funestus*. Mosquitoes were also exposed to PermaNet 2.0 for up to 8 h in time-exposure assays.

**Results:**

Resistance to pyrethroids in *An. funestus s.s.* was extremely high, much higher than reported in 2002 and 2009. No exposure killed more than 25.8% of the mosquitoes tested (average mortality, deltamethrin: 6.4%; lambda-cyhalothrin: 5.1%; permethrin: 19.1%). There was no significant difference in the mortality generated by 3-min exposure to any net (Olyset: 9.3% mortality, PermaNet 2.0: 6.0%, untreated: 2.0%; p = 0.2). Six hours of exposure were required to kill 50% of the *An. funestuss.s.* on PermaNet 2.0.

**Conclusions:**

*Anopheles funestus s.s.* in Manhiça is extremely resistant to pyrethroids, and this area is clearly a pyrethroid-resistance hotspot. This could severely undermine vector control in this district if no appropriate countermeasures are undertaken. The National Malaria Control Programme (NMCP) of Mozambique is currently improving its resistance monitoring programme, to design and scale up new management strategies. These actions are urgently needed, as the goal of the NMCP and its partners is to reach elimination in southern Mozambique by 2020.

**Electronic supplementary material:**

The online version of this article (doi:10.1186/s12936-015-0807-z) contains supplementary material, which is available to authorized users.

## Background

Chemical insecticides are crucial tools in malaria control and elimination programmes. Introduced on walls inside houses as indoor residual sprays (IRS), or on/into the fibers of long-lasting insecticide-treated nets (LLINs), their main purpose is to reduce the risk of human malaria infections by killing the mosquito vectors [1]. Based on World Health Organization (WHO) estimates, these tools have been extremely successful, their scale-up contributing to avert 1.1 million deaths between 2000 and 2012 [2]. However, they also impose a strong selection pressure on the mosquito population for insecticide resistance; there is currently no malaria-affected country in Africa that is free of insecticide-resistant mosquitoes [3]. The malaria community fears that this resistance will undermine effective mosquito control and elimination strategies, and erode the progress that has been made in reducing the number of malaria deaths to-date [2–5].

It is not clear what level of resistance, as defined by current methods and guidelines, precipitates control failure in terms of human malaria cases in an area [6]. For example, a recent systematic review and meta-analysis of the relationship between resistance and control concluded that ITNs remained more effective than untreated nets against resistant mosquitoes, in terms of entomological metrics such as mosquito mortality and blood feeding success [6], though they found only one study that directly compared resistant and susceptible insects [7]. Even with the uncertainty underlying the association, resistance monitoring remains a recommended priority of vector control programmes, so that action can be taken before failure occurs [8, 9].

The National Malaria Control Programme (NMCP) of Mozambique, in collaboration with the WHO, Manhiça Foundation, the President’s Malaria Initiative, USAID and other partners, is currently designing and scaling up activities to eliminate malaria from southern Mozambique by 2020. Insecticidal vector control measures are an important component of the plans for the future, as they have been in the past and are today. In the district of Manhiça, for example, which experiences moderate levels of malaria transmission perennially [10], IRS has been deployed annually since 2005. Mainly DDT and bendiocarb have been provided to the district, although lambda-cyhalothrin (2005 and possibly 2010) and deltamethrin (2009 and 2014) have also been used in IRS campaigns (Additional file 1). LLINs have been handed out to pregnant women at the local antenatal clinics as part of research studies before or, more recently, as national policy for pregnant women. In June–July 2014, a campaign ran in the Manhiça district to achieve universal LLIN coverage, defined as one net per 1.8 persons, and over 32,000 LLINs have been distributed.

Though insecticide use has been widespread, resistance monitoring of the local *Anopheles funestus* population has not been conducted since 2009. An earlier study, in 2002 [11], showed no resistance to permethrin or deltamethrin at the time. In 2009, however, resistance to deltamethrin (52.6% mosquito mortality) and lambda-cyhalothrin (33.3%) was observed [12]. The intensified vector control underway in Manhiça and the surrounding districts will increase insecticidal selection pressure on local mosquito populations to develop insecticide resistance, if no resistance management strategy is in place. To design such strategies, continuous mosquito resistance monitoring and regular evaluations of frontline vector control tool efficacy against local vectors are crucial. To outline the current status of the *An. funestus* population in Manhiça, this work presents (1) the March 2014 levels of resistance to three important different pyrethroids and (2) the impact of resistance on the efficacy of the two common LLIN types found in the district.

## Methods

### Mosquito collections

During February 2014, a total of 778 blood-fed anopheline mosquitoes were collected over a 3-week period from houses in the Ribangua area of Manhiça, close to the irrigated sugar cane fields of Maragra (Illovo) Sugar Mill. IRS teams from the NMCP visit this area annually (for history of IRS in Manhiça, see Additional file 1), but spraying had not yet been done. Females were caught indoors, using mouth aspirator and torch, from 6 am to 12 pm. Before a home was entered, the study was explained in the local language, any questions were answered, and the head of each household gave informed oral consent.

All field-collected females were given the opportunity to oviposit. Because live adults cannot be easily identified to species, a subset was examined by microscopy [13] after eggs were laid. *Anopheles funestuss.l.* dominated the collection, with a few *Anopheles gambiae s.l*. individuals. The species were easily separated as larvae, so the F_1_ generation of each species could be used for bioassays. Larvae were reared to adults at ambient room temperature on a diet of TetraminBaby fish food (Tetra, Germany).

### Resistance bioassays

Two-to-five day-old non-blood-fed female *An. funestus s.l.* mosquitoes were exposed to 0.05% deltamethrin (2 exposures, each with 4 replicates), 0.05% lambda-cyhalothrin (1 exposure with 4 replicates), 0.75% permethrin (2 exposures, each with 4 replicates) for 1 h using WHO-standard insecticide-treated papers, exposure tubes, control papers (silicon oil) and exposure procedures [14]. One-to-eight day-old non-blood-fed female *An. gambiae s.l*. were exposed to 0.05% deltamethrin (two exposures, each with four replicates) in the same way (a larger age range was required to get a sufficient number of individuals). Sample sizes are listed in Table 1. After the exposure, mosquitoes had ad libitum access to sugar water. Mortality was scored 24 h post-exposure. All mosquitoes used in bioassays were identified to species by microscopy [13] and stored individually on silica gel.

In order to maintain constant environmental conditions during and after exposure, mosquitoes were kept in a polystyrene box [inner dimensions 31.5 × 35 × 22(h) cm] outfitted with heat cable (PT2012 Heat Cable, ExoTerra, USA) and a humidifier (RF-10E ReptiFogger, ZooMed, USA). Temperature and relative humidity (RH) were held at 26 ± 1°C and 90 ± 10% RH by a thermostat/humidistat (HT-10E Hygrotherm, ZooMed, USA) and were recorded at 5 min intervals by an automated logger (OM-62, Omega, Spain).

To authenticate the quality of the insecticide-treated papers, two-to-five day-old laboratory-reared susceptible *Anopheles arabiensis* females were exposed in a similar fashion. These experiments were performed at the insectaries of the National Institute of Health in Mozambique, at 27 ± 2°C and 80 ± 10% RH.

### Long-lasting insecticide-treated net tests

The efficacy of two previously unopened Olyset (permethrin LLIN, manufactured 11/2010, obtained from the Manhiça Health Research Centre) and two previously unopened PermaNet 2.0 bed nets (deltamethrin LLIN, 05/2011, purchased in local store) was evaluated using two-to-five day-old, non-blood-fed, female *An. funestus s.l.* in standard WHO cone bioassays [9]. Groups of five mosquitoes were exposed for 3 min to pieces of netting (four pieces cut from sides of each net tested; Olyset: n = 97 mosquitoes, PermaNet 2.0: n = 96). A locally purchased untreated bed net (China Da Hua Shoes, LDA Moçambique), also previously unopened, served as control netting (n = 49). Mosquitoes were exposed to nets at 26 ± 2°C and 60 ± 10% RH, and then transferred to cups and maintained in the polystyrene box described above at 26 ± 1°C and 90 ± 10% RH for 24 h, after which mortality was recorded. To confirm the insecticidal activity of the LLINs, wild-caught blood-fed *Anopheles tenebrosus* females (abundant in our study area) were tested in a similar fashion. All mosquitoes used in the bioassays were identified to species by microscopy [13] and stored individually on silica gel.

In order to evaluate the level of resistance to LLINs, mosquitoes were also exposed to LLINs for longer durations than the 3 min advised in the WHO protocol (Experiment 1: 3 min, 1, 2, 4, 6 h; experiment 2: 3 min, 2, 4, 6, 8 h). In each experiment, for each time treatment, two replicates of five mosquitoes were exposed to untreated netting and four replicates of five mosquitoes to LLIN. Only the two PermaNet 2.0 LLINs were tested in time-exposure experiments, as mosquitoes could be caught beneath the larger mesh openings of the Olyset nets, making mortality rates for longer durations of exposure unreliable.

### Mosquito identification

Forty-eight *An. gambiae s.l*. and two hundred *An. funestuss.l.* F_1_ females were identified to species by PCR using the methods of Scott et al. [15] and Koekemoer et al. [16], respectively. Extraction of DNA was done for the *An. funestus* group, and a single leg was used directly as template for the *An. gambiae* complex. Individuals identified as *An. arabiensis* were screened for the presence of East- and West-knockdown resistance (*kdr*) alleles using the methods of Bass et al. [17].

### Data analysis

‘Resistance’ to a given chemical or net was designated according to the current WHO criteria, based on the percent mortality observed 24 h after insecticide exposure: mosquito populations are classified as resistant if more than 2% of the exposed individuals survive [18]. Following the classifications proffered by Strode et al. [6], resistance to a given pyrethroid could be described as ‘low,’ if exposure led to mortality greater than 80%, ‘moderate’ if mortality was between 25 and 80%, and ‘high’ if less than 25% of females were killed by exposure.

Statistical analyses were performed in R v. 2.10.1 [19]. Mortality data were analysed as Generalized Linear Models using a binomial error distribution and logit link function; in cases of overdispersion, the quasibinomial error distribution was used. For tube bioassay analyses, insecticide exposure (control or chemical) was the only independent variable. For the 3-min cone bioassay analyses, net (control, Olyset, or PermaNet 2.0) was the only independent variable. When investigating the strength of resistance to net exposure, duration of exposure (Experiment 1: 3 min, 1, 2, 4, 6 h; experiment 2: 3 min, 2, 4, 6, 8 h) was included as an independent variable as well as net (control or PermaNet 2.0). Pieces from two different PermaNets were used and the results from the two experiments were pooled, so net replicate and experiment were also included in the model as random effects. Maximal models were fitted with random effects and interaction terms first, and then non-significant terms were removed by backward-elimination.

## Results

### *Anopheles funestus*

**Fig. 1:**
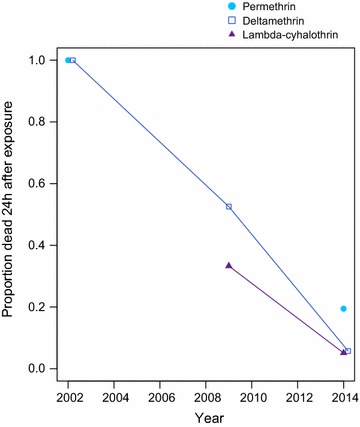
Observed increase in pyrethroid resistance in *Anopheles funestus* mosquitoes in Manhiça, as measured by decreased mortality after exposure. From undetectable levels in 2002, resistance to deltamethrin and permethrin has increased drastically; in early 2014, less than one-third of exposed mosquitoes were killed by an hour-long exposure to either chemical. Resistance to lambda-cyhalothrin has reached similar levels, increasing from 40% to almost complete resistance since 2009 [11; this study, 12].

All PCR-identified individuals were *An. funestuss.s*. (181/200; 19 did not amplify), so it is assumed that the results pertain to this species. WHO tube tests (Table 1) revealed that *An.funestus* was highly resistant to deltamethrin (3.1 and 9.6% mortality, average: 6.4%) and permethrin (13.4 and 25.8% mortality, average: 19.1%). There was also clear resistance to lambda-cyhalothrin, as only 5.1% of the 78 mosquitoes were killed [18]. When compared to previous similar resistance surveys in the area [11, 12], resistance to pyrethroids in *An. funestus* has emerged and spread rapidly over the past 12 years (Fig. 1).

**Fig. 2:**
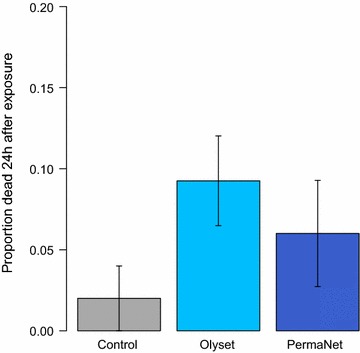
Mortality of *Anopheles funestus* 24 h after 3-min exposure to LLINs or untreated bed nets. Exposure to Olyset (permethrin, n = 97) or PermaNet 2.0 (deltamethrin, n = 96) nets did not kill significantly more mosquitoes than an untreated bed net (n = 49; *p* = 0.2).

When *An. funestus* was exposed to LLINs incorporating permethrin (Olyset) or deltamethrin (PermaNet 2.0), mortality after a standard exposure of 3 min did not differ from exposure to an untreated net (Mean ± SEM, Olyset: 9.3 ± 2.8%, PermaNet 2.0: 6.0 ± 3.3%, untreated: 2.0 ± 2.0%; Fig. 2; X^2^ = 3.3, df = 2, *p* = 0.2). In contrast, 94.7% (18/19) of wild-caught, blood-fed *An. tenebrosus* females were killed after 3-min exposure (5.6% mortality in the control group, 1/18).

**Fig. 3:**
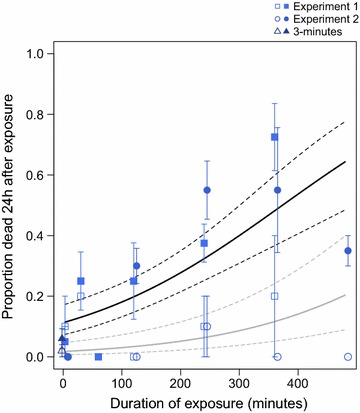
Time-mortality relationship for *Anopheles funestus* mosquitoes from Manhiça, exposed to PermaNet 2.0 LLINs for durations between 3 min and 8 h. *Filled symbols* indicate mortality from PermaNet exposures, and mortality from untreated nets is indicated by *open symbols* (±1 SEM); experiment 2 points are slightly offset at each timepoint to improve visibility. Model predictions are traced by *solid lines* (*gray* = untreated net, *black* = PermaNet), with *dashed lines* at 95% prediction intervals.

Given the high levels of resistance in the WHO tube tests and the low mortality rates on LLINs, the strength of resistance against PermaNet 2.0 LLINs was assessed by time-exposure assays. PermaNet 2.0 exposure induced greater mortality than untreated nets as the duration of exposure increased (duration of exposure, F = 35.5, df = 1,94, p < 0.001; net treatment, F = 23.5 df = 1,94, p < 0.001; duration × treatment, F = 3.8, df = 1,93, p = 0.06). The random effects of experiment and net replicate were not significant and were excluded from the model (net replicate, F = 1.7, df = 2,92, p = 0.2; experiment, F = 2.0, df = 2,90 p = 0.1). The estimated median lethal time, or LT_50_, is 6 h 13 min (Fig. 3).

### *Anopheles gambiae*

Ninety percent of the *An. gambiae s.l.* (43/48) were identified as *An. arabiensis*, the remaining ten percent (5/48) as *Anopheles merus*. *Anopheles gambiae s.l.* mosquitoes in the area were resistant to deltamethrin, with 89.7–97.5% (mean = 93.6%) killed by 1-h exposure (Table 1). Neither East nor West *kdr* alleles were detected in the *An. arabiensis* tested (0/43). The susceptible *An. arabiensis* colony used as a WHO kit control needs to be tested further, as some baseline resistance to pyrethroids was detected in this insect line.

**Table 1:** Pyrethroid susceptibility of F1 generation *Anopheles* *funestus* and *An. gambiae s.l.* from Manhiça, as well as a laboratory colony of susceptible *An. arabiensis*

Insecticide	Percent mortality (n)	
	Treated	Control
*An. funestus s.s.*		
0.05% Deltamethrin [1]	3.1% (97)	0% (47)
0.05% Deltamethrin [2]	9.6% (94)	0% (47)*
0.75% Permethrin [1]	25.8% (93)	0% (47)*
0.75% Permethrin [2]	12.4% (97)	0% (53)
0.05% Lambda-cyhalothrin	5.1% (78)	1.9% (52)
*An. gambiae s.l.*		
0.05% Deltamethrin [1]	89.7% (29)	0% (25)
0.05% Deltamethrin [2]	97.5% (40)	3.8% (26)
*An. arabiensis*		
0.05% Deltamethrin	100% (54)	3.8% (52)^†^
0.75% Permethrin	90.7% (56)	3.8% (52)^†^
0.05% Lambda-cyhalothrin	94.3% (53)	3.8% (52)^†^

Percentage indicates percent mortality 24 h following 1 h exposure; number between parentheses indicates the number of mosquitoes tested.

Number inside square braces indicates experimental replicate.

* Shared control.

^†^Shared control.

## Discussion

In the last 5 years, pyrethroid resistance in the *An. funestus* population of Manhiça has increased to extremely high levels, and this area in southern Mozambique is clearly a pyrethroid-resistance hotspot (Fig. 1; [12]). Over 90% of mosquitoes survived a deltamethrin or lambda-cyhalothrin exposure, and almost three-quarters of mosquitoes survived an exposure to permethrin (Table 1). Mosquitoes were equally as likely to die from 3-min exposure to a net that was not treated with insecticide as from exposure to a pyrethroid-treated Olyset (permethrin) or PermaNet 2.0 (deltamethrin) (Fig. 2). In addition, the deltamethrin LLIN was unable to kill more than half of the mosquitoes in 6 h. Given that mosquitoes are unlikely to rest on a bed net, undisturbed, for 6 h, these results indicate that the current pyrethroid-based vector control tools are unlikely to effectively kill the major malaria vector in this part of Mozambique.

Due to limited numbers, the local *An. gambiae s.l.* was only screened for resistance to deltamethrin. These mosquitoes, a mix of ninety percent *An. arabiensis* and ten percent *An. merus*, were resistant, with ten percent surviving deltamethrin exposure (note that this ten percent includes both species). The level of resistance appears to be much lower than in *An. funestus*, in which around ninety percent of mosquitoes survived. This difference is interesting, given that the field-caught females that generated these experimental populations were captured in ostensibly identical locations, indicating some overlap in their behaviour and ecology and, therefore, some shared selection pressures. For resistance management strategies to have any hope of being successful, it is critical that the major selection pressures at work in a population be identified. In a transmission setting with multiple vector species, differences in the ecology and the biology of those species (such as differences in biting time, resting habits, or larval habitats) could prove to be important.

Even in the face of insecticide resistance, LLINs will continue to play an important role in malaria control programmes. Well-maintained LLINs still provide a physical barrier between mosquitoes and human hosts and can prevent bites regardless of insecticidal activity [20]. When pyrethroids are no longer as deadly to resistant vectors, they can still act as repellents and/or irritants [6, 21]. These effects could interfere with disease transmission even when no mosquitoes are killed by an insecticide (described in [1]). However, to increase the impact of vector control strategies, alternative LLINs, such as the Olyset Plus and PermaNet^®^ 3.0 should be tested against the local *An. funestus* populations. In addition to a pyrethroid, both of these nets include the pyrethroid-synergist piperonyl-butoxide (PBO), which inhibits the activity of metabolic detoxification enzymes [22]. These nets have demonstrated efficacy against pyrethroid-resistant mosquitoes [23, 24], and two studies indicate that such LLINs may be effective in the Manhiça area [12, 25].

While these alternative LLINs could improve the mosquito control generated by bed nets, they still can only be treated with pyrethroid insecticides [26, 27] and, thus, will not remove the selection pressure for pyrethroid resistance. Control of the highly-pyrethroid-resistant mosquitoes in Manhiça, therefore, will rely on the use of non-pyrethroid chemicals for IRS. Unfortunately, high levels of resistance to the carbamate bendiocarb ([12] and Additional file 2) have also been detected in the area, which limits our chemical arsenal to DDT ([12] and Additional file 2) or pirimiphos-methyl (Additional file 1), and perhaps malathion [12], but additional, systematic evaluations are needed.

Together, the high levels of pyrethroid resistance detected in standard bioassays and the duration of LLIN exposure necessary to kill *An. funestus* from Manhiça seen here indicate that vector control plans for southern Mozambique will have to carefully integrate multiple control tools in order to achieve effective malaria control in this pyrethroid resistance ‘hot spot’ [5]. The NMCP of Mozambique is aware of the pressing issue of pyrethroid resistance, is currently improving its resistance-monitoring programme and is drafting a resistance management strategy to counteract the rising problem of insecticide resistance. As mosquito control, resistance-monitoring and –management programmes proceed in this area, it will be important to consistently evaluate their impact, in order to maintain effective control and provide useful information to the other countries pushing toward malaria elimination in the coming decades.

## Additional files

Additional file 1: Insecticides used for indoor residual spraying in the Manhiça area, 2005–2014. Table of local IRS history Additional file 2: Insecticide susceptibility of F1 generation *Anopheles* *funestus* from Manhiça, as well as a laboratory colony of susceptible *An. arabiensis*. Table of results from exposures to non-pyrethroid insecticides

## Abbreviations

IRS: indoor residual spraying
ITNs: insecticide-treated nets
LLINs: long-lasting insecticide-treated nets
NMCP: National Malaria Control Programme
RH: relative humidity
WHO: World Health Organization
WHOPES: World Health Organization Pesticide Evaluation Scheme

## References

[1] Pates H, Curtis C (2005). Mosquito behavior and vector control. Annu Rev Entomol.

[2] WHO (2012) World Malaria Report 2012. World Health Organization, Geneva. http://www.who.int/malaria/publications/world_malaria_report_2012/en/

[3] Hemingway J (2014). The role of vector control in stopping the transmission of malaria: threats and opportunities. Philos Trans R Soc Lond B Biol Sci.

[4] Maxmen A (2012). Malaria surge feared. Nature.

[5] WHO (2012). Global plan for insecticide resistance management in malaria vectors.

[6] Strode C, Donegan S, Garner P, Enayati AA, Hemingway J (2014). The impact of pyrethroid resistance on the efficacy of insecticide-treated bed nets against African anopheline mosquitoes: systematic review and meta-analysis. PLoS Med.

[7] Chandre F, Darriet F, Duchon S, Finot L, Manguin S, Carnevale P (2000). Modifications of pyrethroid effects associated with *kdr* mutation in *Anopheles gambiae*. Med Vet Entomol.

[8] Global Malaria Programme (2010). Meeting report: the technical basis for coordinated action against insecticide resistance: preserving the effectiveness of modern malaria vector control.

[9] WHO (2013). Test procedures for insecticide resistance monitoring in malaria vector mosquitoes.

[10] Moncunill G, Mayor A, Bardají A, Puyol L, Nhabomba A, Barrios D (2013). Cytokine profiling in immigrants with clinical malaria after extended periods of interrupted exposure to *Plasmodium falciparum*. PLoS One.

[11] Casimiro S, Coleman M, Mohloai P, Hemingway J, Sharp B (2006). Insecticide resistance in *Anopheles funestus* (Diptera: Culicidae) from Mozambique. J Med Entomol.

[12] Kloke RG, Nhamahanga E, Hunt RH, Coetzee M (2011). Vectorial status and insecticide resistance of *Anopheles funestus* from a sugar estate in southern Mozambique. Parasit Vectors.

[13] Gillies M, Coetzee M (1987). A supplement to the anophelinae of Africa south of the Sahara. Publ S Afr Inst Med Res.

[14] WHO (2006). Guidelines for testing mosquito adulticides for indoor residual spraying and treatment of mosquito nets.

[15] Scott JA, Brogdon WG, Collins FH (1993). Identification of single specimens of the *Anopheles gambiae* complex by the polymerase chain reaction. Am J Trop Med Hyg.

[16] Koekemoer L, Kamau L, Hunt R, Coetzee M (2002). A cocktail polymerase chain reaction assay to identify members of the *Anopheles funestus* (Diptera: Culicidae) group. Am J Trop Med Hyg.

[17] Bass C, Nikou D, Donnelly MJ, Williamson MS, Ranson H, Ball A (2007). Detection of knockdown resistance (*kdr*) mutations in *Anopheles gambiae*: a comparison of two new high-throughput assays with existing methods. Malar J.

[18] WHO (2013). Guidelines for laboratory and field-testing of long-lasting insecticidal nets.

[19] R Development Core Team (2008). R: a language and environment for statistical computing.

[20] Okumu FO, Mbeyela E, Lingamba G, Moore J, Ntamatungiro AJ, Kavishe DR (2013). Comparative field evaluation of combinations of long-lasting insecticide treated nets and indoor residual spraying, relative to either method alone, for malaria prevention in an area where the main vector is *Anopheles arabiensis*. Parasit Vectors.

[21] Grieco JP, Achee NL, Chareonviriyaphap T, Suwonkerd W, Chauhan K, Sardelis MR (2007). A new classification system for the actions of IRS chemicals traditionally used for malaria control. PLoS One.

[22] Feyereisen R (1999). Insect P450 enzymes. Annu Rev Entomol.

[23] Corbel V, Chabi J, Dabiré RK, Etang J, Nwane P, Pigeon O (2010). Field efficacy of a new mosaic long-lasting mosquito net (PermaNet^®^ 3.0) against pyrethroid-resistant malaria vectors: a multi centre study in Western and Central Africa. Malar J.

[24] Pennetier C, Bouraima A, Chandre F, Piameu M, Etang J, Rossignol M (2013). Efficacy of Olyset^®^ Plus, a new long-lasting insecticidal net incorporating permethrin and piperonyl-butoxide against multi-resistant malaria vectors. PLoS One.

[25] Brooke B, Kloke G, Hunt R, Koekemoer L, Tem E, Taylor M (2001). Bioassay and biochemical analyses of insecticide resistance in southern African *Anopheles funestus* (Diptera: Culicidae). Bull Entomol Res.

[26] WHO (2014) Recommended long-lasting insecticidal nets. World Health Organization, Geneva. http://www.who.int/whopes/Long_lasting_insecticidal_nets_06_Feb_2014.pdf

[27] WHOPES (2007) Insecticides for ITNs. World Health Organization, Geneva. http://www.who.int/whopes/en/

