# Supplementary material for: Long-lasting insecticidal nets no longer effectively kill the highly resistant Anopheles funestus of southern Mozambique
Source: Malar J. 2015 Aug 5;14:298. doi: 10.1186/s12936-015-0807-z (PMC4524426; doi:10.1186/s12936-015-0807-z)
Supplement: Additional file 1: — Insecticides used for indoor residual spraying in the Manhiça area, 2005–2014. Table of local IRS history [file 12936_2015_807_MOESM1_ESM.docx]

| **Period** | **Insecticide** |
| --- | --- |
| **Manhiça**^*^ |  |
| 2005 - 2006 | Lambda-cyhalothrin |
| 2006 - 2008 | DDT, bendiocarb |
| 2009 | DDT, bendiocarb, deltamethrin |
| 2010 | DDT, bendiocarb, lambda-cyhalothrin^**^ |
| 2011 - 2012 | DDT, bendiocarb |
| 2014 | Bendiocarb, deltamethrin |
| **Maragra**^†^ |  |
| 1996 - 2000 | Propoxur |
| 2000 - 2012 | Bendiocarb |
| 2012 - date | DDT and pirimiphos-methyl |
|  |  |

^*^Data obtained from National Malaria Control Programme

^**^No data for Manhiça, insecticides distributed to Maputo Province

^†^Personal communication Eduardo Nhamahanga, Maragra (Illovo) Sugar Estate. Houses at the edge of Manhiça near the Sugar Estate are sprayed by the company, independent of the NMCP.

**Additional file 1** **Insecticides used for indoor residual spraying in the Manhiça area, 2005-2014**
