# Supplementary material for: Long-lasting insecticidal nets no longer effectively kill the highly resistant Anopheles funestus of southern Mozambique
Source: Malar J. 2015 Aug 5;14:298. doi: 10.1186/s12936-015-0807-z (PMC4524426; doi:10.1186/s12936-015-0807-z)
Supplement: Additional file 2: — Insecticide susceptibility of F1 generation Anopheles funestus from Manhiça, as well as a laboratory colony of susceptible An. arabiensis. Table of results from exposures to non-pyrethroid insecticides [file 12936_2015_807_MOESM2_ESM.docx]

^^ ^*^Shared control, ^†^Papers treated with olive oil instead of Risella oil

**Additional file 2** **Insecticide susceptibility of F1 generation *Anopheles* *funestus* from Manhiça, as well as a laboratory colony of susceptible *An. arabiensis***

Two-to-five day old female mosquitoes were exposed to control or insecticide-treated papers for one hour, according to the standard WHO tube bioassay procedures. Percentage indicates mortality 24 hours following exposure, and the number between parentheses indicates the number of mosquitoes tested.
